# An update on the mechanical versus manual cardiopulmonary resuscitation in cardiac arrest patients

**DOI:** 10.1186/s13054-024-05131-7

**Published:** 2024-10-22

**Authors:** Ayman El-Menyar, Mashhood Naduvilekandy

**Affiliations:** 1 Clinical Research, Trauma and Vascular Surgery, Hamad Medical Corporation, Doha, Qatar; 2 Department of Medicine, Weill Cornell Medicine, Doha, Qatar

## To the editor

**Fig. 1:**
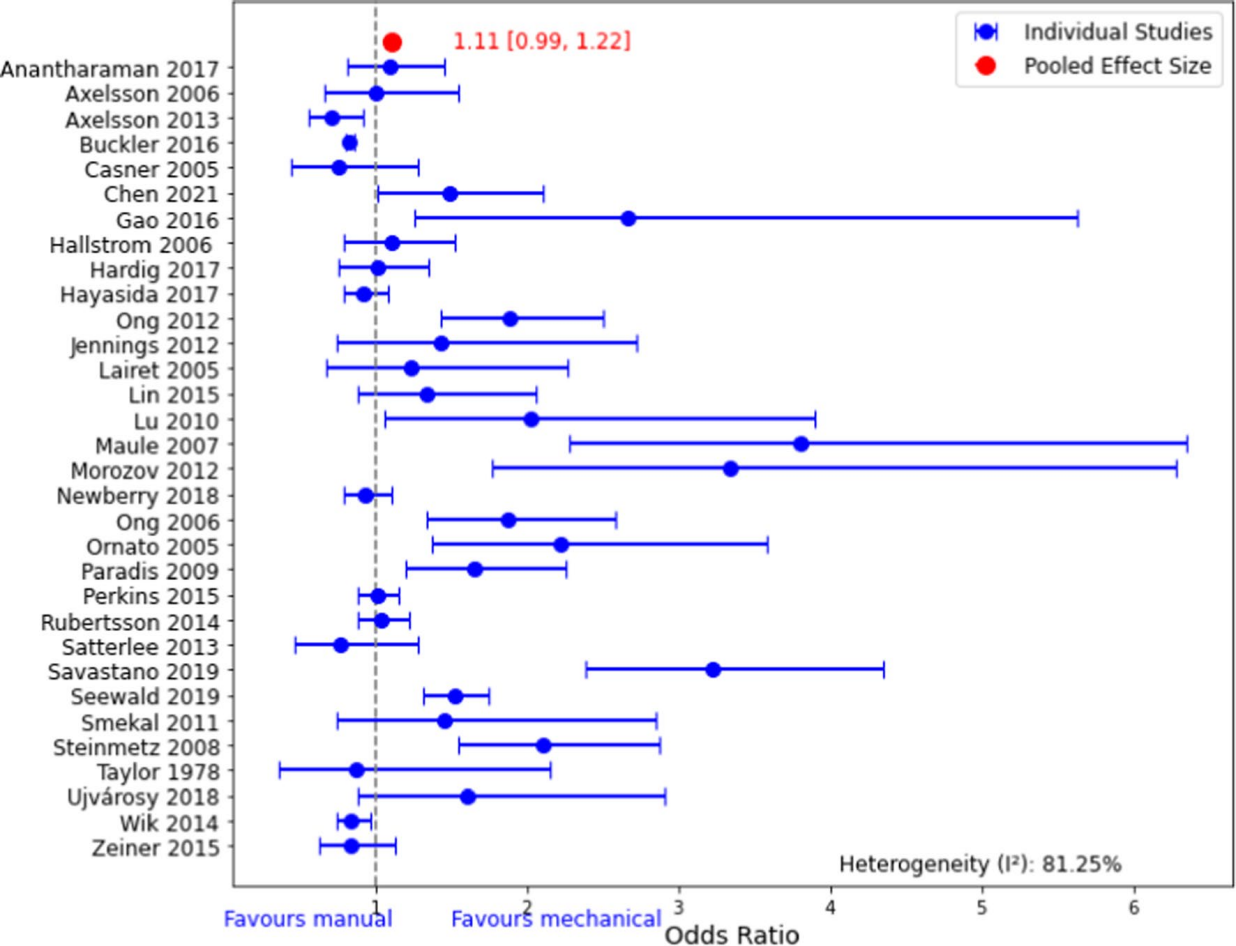
Revised Forest plot of pooled odds ratio for ROSC of studies included in the Umbrella review after removing the duplicate and a study with a high variance

The cardiopulmonary resuscitation (CPR) technique and its outcome remains a debate. In response to Zhao et al.'s [1] regarding the inclusion of duplicated studies in the meta-analysis [2], we have conducted a thorough review of the two studies published by Ong et al. (2012) and Casner et al. (2005). After that, we removed these two studies, along with an additional data point from Halperin et al. (1993), which exhibited high variance and did not meet the variance thresholds set for our updated analysis, and then we performed a revised meta-analysis to maintain consistency. Despite these changes, the results remained consistent, with an (Odds Ratio (OR) of 1.11; 95% CI 0.99–1.22) (Fig. 1). Thus, our original umbrella review findings [2] and Zhao et al.'s analysis showed that mechanical CPR was not superior to manual CPR in achieving the return of spontaneous circulation (ROSC).

We respectfully disagree with Zhao et al. second point regarding the inclusion of Axelson et al. (2013) and Jennings et al. (2012) in the meta-analysis. A few relevant data required for our analysis were obtained from the already published systematic review (SR) by Sheraton et al. (2021) [3]. The ROSC-related ORs were extracted from the second graph of the Sheraton et al. meta-analysis [3]. It is also worth noting that Zhao et al. [1] included a study published by Hallstrom et al. (2006) [4], even though this study did not explicitly mention ROSC as an outcome in the original work.

**Fig. 2:**
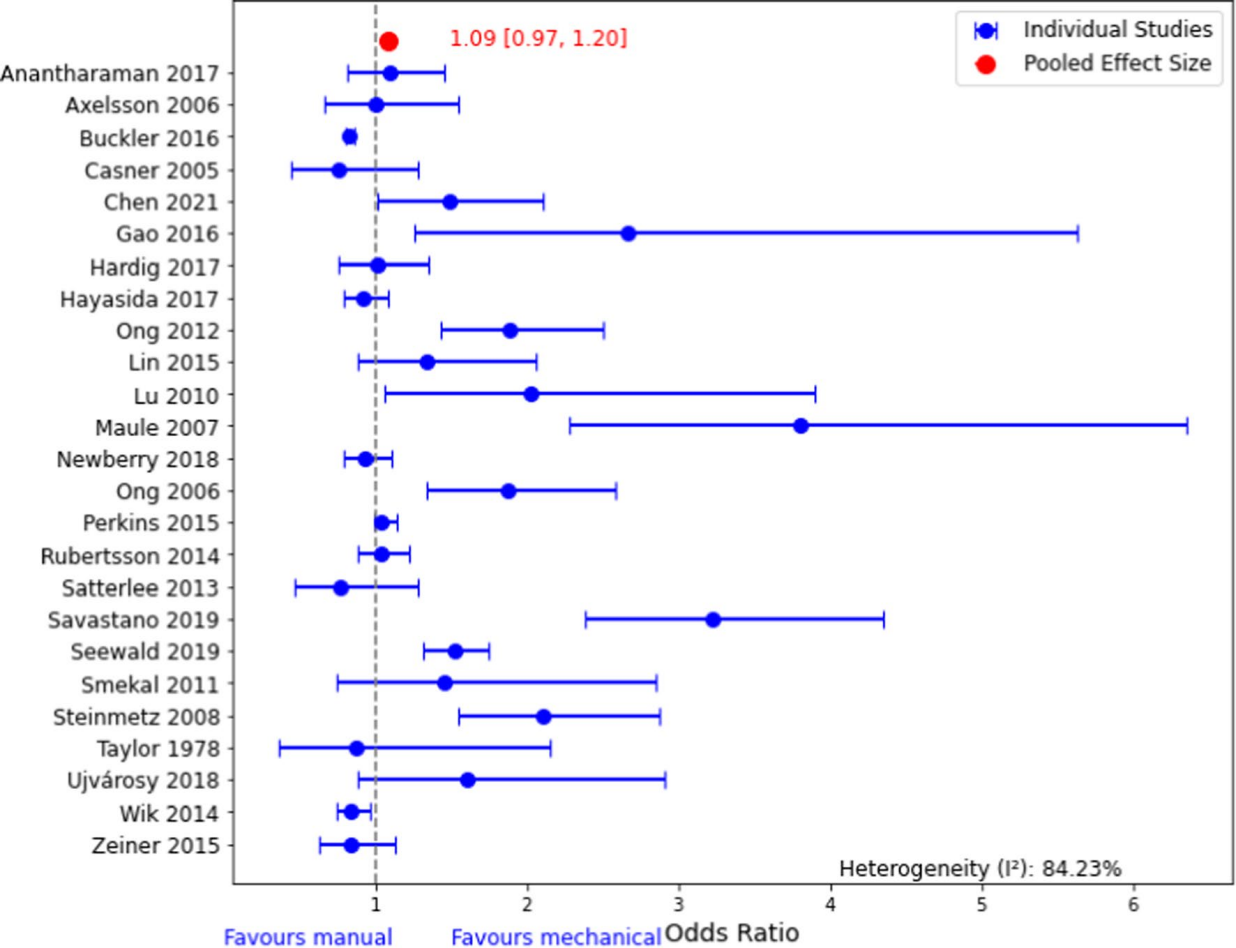
Revised Forest plot of the pooled odds ratio of ROSC without data from abstracts or studies did not report ROSC as an outcome in their original research

We agree that data derived solely from abstracts can affect the robustness of outcomes; therefore, we intended to gather and synthesize as much data as possible from the published SRs and not from individual studies or abstracts for the umbrella review [2]. However, for the umbrella meta-analysis, data from 3 abstracts (Lairet et al. (2005), Paradis et al. (2009), and Morozov et al. (2012)) were used. The ORs from these abstracts were extracted from a meta-analysis published by Bonnes et al. (2016) and their illustrations [5]. Thus, in this letter, we recalculated the ORs after removing data gathered from abstracts or did not report ROSC-related ORs in the original works. The results of the revised analysis did not show any significant difference (OR 1.09; 95% CI 0.97–1.20) compared to our previous results (OR 1.05; 95% CI 0.94–1.15) [2] (Fig. 2).

We also disagree with Zhao et al. on their concern for inclusion of Couper et al. (2021) Randomized Clinical Trial (RCT) [6]. Our previous meta-analysis [2] focused on publications from April 2021 to February 2024; this RCT was published in January 2021. Therefore, it fell outside our search period and was not included in our new SR and meta-analysis.

The survival rate post-cardiac arrest varies according to several factors, including the location of arrest (in-hospital (IHCA) vs out-of-hospital (OHCA) cardiac arrest), time to ROSC, and the impact of the post-cardiac arrest myocardial dysfunction or syndrome. After IHCA, the survival is almost twice that of OHCA, as an early ROSC is highly attained after IHCA (≈ 50%) [7]. Zhao et al. results were consistent with our findings, showing that in patients with OHCA, mechanical CPR did not improve the ROSC in RCTs and non-RCTs, while after IHCA, RCTs showed improved ROSC with mechanical CPR. However, the subgroup analysis for the IHCA group included very old RCTs (two out of four, namely Taylor 1978 and Halperin 1993), in addition to the comparatively high variances [1]. It is essential to highlight those significant changes in the resuscitation protocols and advancements in technology that have occurred over the past decades. The age of these studies presents a considerable challenge, as they were conducted under vastly different clinical environments and standards of care compared to more recent studies. These variations, including differences in CPR techniques, medications, and post-resuscitation care, further contribute to the heterogeneity and bias of the outcomes data.

It is of utmost importance that the data used in our umbrella review and SR showed a heterogeneity due to the different methodologies employed in the original studies and SRs. The design and quality of studies were also of concern [2]. Moreover, the definitions of ROSC and the cause of cardiac arrest varied across studies, introducing additional complexity to the analysis. We meticulously addressed these issues in our review including a comprehensive limitations section for the readers [2].

Of note is that the likelihood of ROSC and survival is expected to significantly improve when CPR is performed promptly and at a high-quality level using advanced technology and guidelines. Despite advances in CPR, poor survival rates remain challenging, even with achieving the ROSC. Therefore, ROSC should not be the end of the game, and research must also focus on preventing the post-cardiac arrest syndrome that could occur within several hours of the arrest and ROSC [7]. A recent SR (24 studies) showed no statistically significant differences in ROSC and survival between the two kinds of CPR following OHCA. However, a favorable neurological outcome (OR 1.41; 95% CI 1.07–1.84) was observed with manual CPR in 13 of these OHCA studies [8]. Therefore, we addressed these essential outcomes after CPR along with the ROSC, such as survival to hospital admission, survival to hospital discharge, 30-day survival, and neurological outcomes. Additionally, we investigated the impact of gender, age, type of device, initial rhythm, duration of CPR, and location of arrest on the CPR outcomes in subgroups, ensuring comprehensive and robust analysis. Consequently, we urge well-designed multicenter RCT and living SR and umbrella review to support favorable post-CPR outcomes and overcome the gaps in contemporary literature.

## Data Availability

No datasets were generated or analysed during the current study.
